# Management of Extensive Hepatic Metastases From Adenoid Cystic Carcinoma of the Parotid Gland by Embolization and Hemi-Hepatectomy: A Case Report

**DOI:** 10.7759/cureus.81860

**Published:** 2025-04-07

**Authors:** Gunjan Desai, Kaustubh Lokhande, Deepak Parikh, Prasad Wagle

**Affiliations:** 1 Gastrointestinal Surgery, Lilavati Hospital and Research Centre, Mumbai, IND; 2 Radiology, Lilavati Hospital and Research Centre, Mumbai, IND; 3 Head and Neck Surgery, Asian Cancer Institute and ACI Cumballa Hill Hospital, Mumbai, IND; 4 Surgical Gastroenterology, Lilavati Hospital and Research Centre, Mumbai, IND

**Keywords:** adenoid cystic carcinoma (acc), metastatic liver cancer, parotid tumours, portal vein embolization, rare liver mass, right hepatectomy

## Abstract

Adenoid cystic carcinoma (ACC) is a rare malignancy of the salivary glands characterized by slow growth, perineural invasion, and a predilection for distant metastasis, primarily to the lungs and bones. Liver metastasis from ACC is uncommon and poses significant diagnostic and therapeutic challenges. This report presents a case of mixed type of ACC (predominantly cribriform and tubular architecture with a solid component of 20%) originating in the parotid gland with multiple liver metastases from a tertiary center in India. A 32-year-old woman initially underwent a total conservative parotidectomy with facial nerve preservation and adjuvant radiotherapy for ACC of the right parotid gland in 2017. Immunohistochemistry was positive for CD117, S100, and smooth muscle actin (SMA). Following a recurrence of metastatic neck lymph node involvement in 2020, she underwent right modified radical neck dissection and additional radiation therapy. In 2024, surveillance imaging revealed liver lesions, confirmed as ACC metastases by biopsy with immunohistochemistry. Due to inadequate hepatic reserve for a right hemi-hepatectomy, a right portal vein embolization was performed to induce hypertrophy of the left liver. Subsequent right hemi-hepatectomy with segment II metastatectomy achieved an R0 resection, and the patient experienced an uneventful recovery. She remained disease-free six months postoperatively. ACC accounts for 1%-2% of all head and neck cancers and exhibits distinct clinical and pathological features. Its treatment is centered on surgical resection with adjuvant radiotherapy, although the efficacy of chemotherapy is limited. Liver metastasis, though rare, can be managed with surgical intervention in selected cases, potentially improving survival outcomes. This case underscores the importance of long-term follow-up for ACC patients due to the risk of delayed distant metastases. It also highlights the role of multidisciplinary management and individualized treatment strategies in achieving optimal outcomes in rare and complex clinical scenarios.

## Introduction

Pleomorphic adenoma is the most common benign parotid tumor, while mucoepidermoid carcinoma is the most common malignant parotid neoplasm. Adenoid cystic carcinoma (ACC) is a rare malignant neoplasm of the salivary glands, characterized by slow growth and a propensity for peri-neural invasion. It often presents with painless swelling, but its aggressive nature and propensity for distant metastasis make early diagnosis and treatment critical [1]. Lymph node metastasis in ACC is uncommon, but when present, it indicates a more aggressive disease course and poorer prognosis. While ACC commonly metastasizes to the lungs and bones, liver metastasis is notably uncommon, presenting unique challenges in diagnosis and management. Despite its slow progression, metastatic ACC poses significant challenges due to its resistance to conventional therapies and limited treatment options [2]. In this report, we present a case of ACC from the parotid gland with multiple liver metastases managed at our specialized hepatobiliary unit and provide a relevant literature review of this rare hepatic malignancy.

## Case presentation

A 32-year-old female with no prior medical history presented in 2017 with a mass in the right parotid region, subsequently diagnosed as ACC. She underwent total conservative parotidectomy with facial nerve preservation followed by adjuvant radiation therapy. The histology in the index surgery was a mixed pattern of ACC with predominant cribriform and tubular components and a solid component of 20%. Immunohistochemistry was positive for CD117, smooth muscle actin (SMA), and S100. The margins of resection were free from the tumor (R0 resection). She had no neck lymphadenopathy, and hence, a neck dissection was not performed at the time.

In 2020, she developed metastatic right neck lymph node involvement, for which a right modified radical neck dissection was performed, followed by radiation therapy. She was on three monthly follow-ups after the surgery with clinical checkups focused on head and neck examination and cranial nerve assessment, annual chest CT scans, as well as an ultrasound abdomen for two years, and after that, a yearly positron emission tomography-computed tomography (PET-CT) follow-up was carried out.

**Figure 1:**
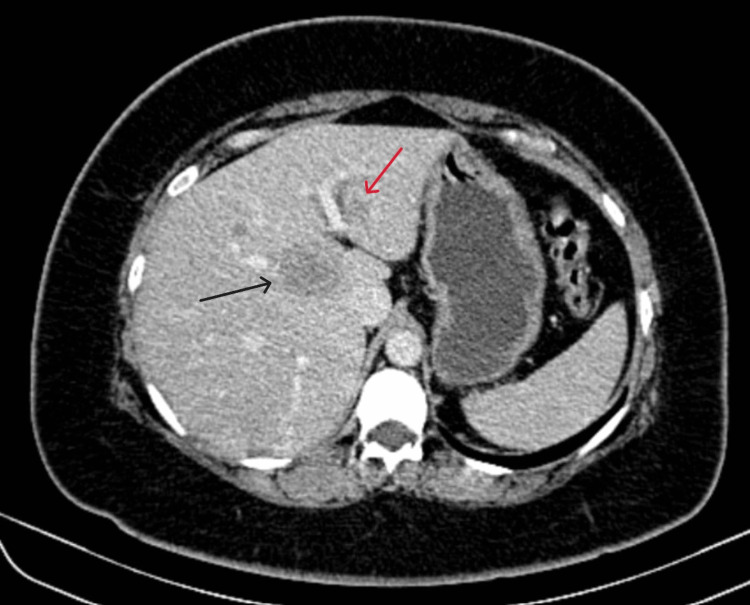
Contrast-enhanced computed tomography scan portal phase axial cut showing a lesion in segment 2 (red arrow) and a lesion in the caudate segment (black arrow) of the liver.

**Figure 2:**
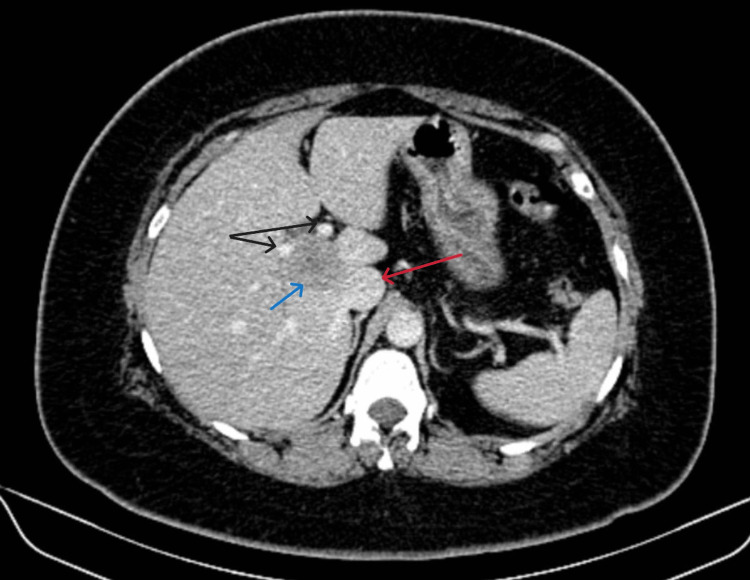
Contrast-enhanced computed tomography scan portal phase axial cut showing a lesion in the caudate segment (blue arrow) of the liver. The caudate relationships can be appreciated in this figure with the poral vein bifurcation anteriorly (black arrow) and inferior vena cava posteriorly (red arrow).

**Figure 3:**
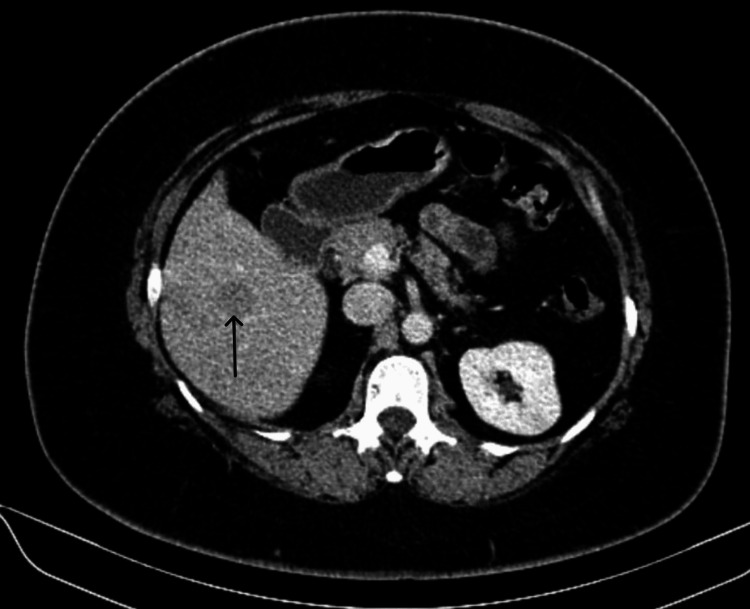
Contrast-enhanced computed tomography scan portal phase axial cut showing a lesion in the segment 6 (black arrow) of the liver.

**Figure 4:**
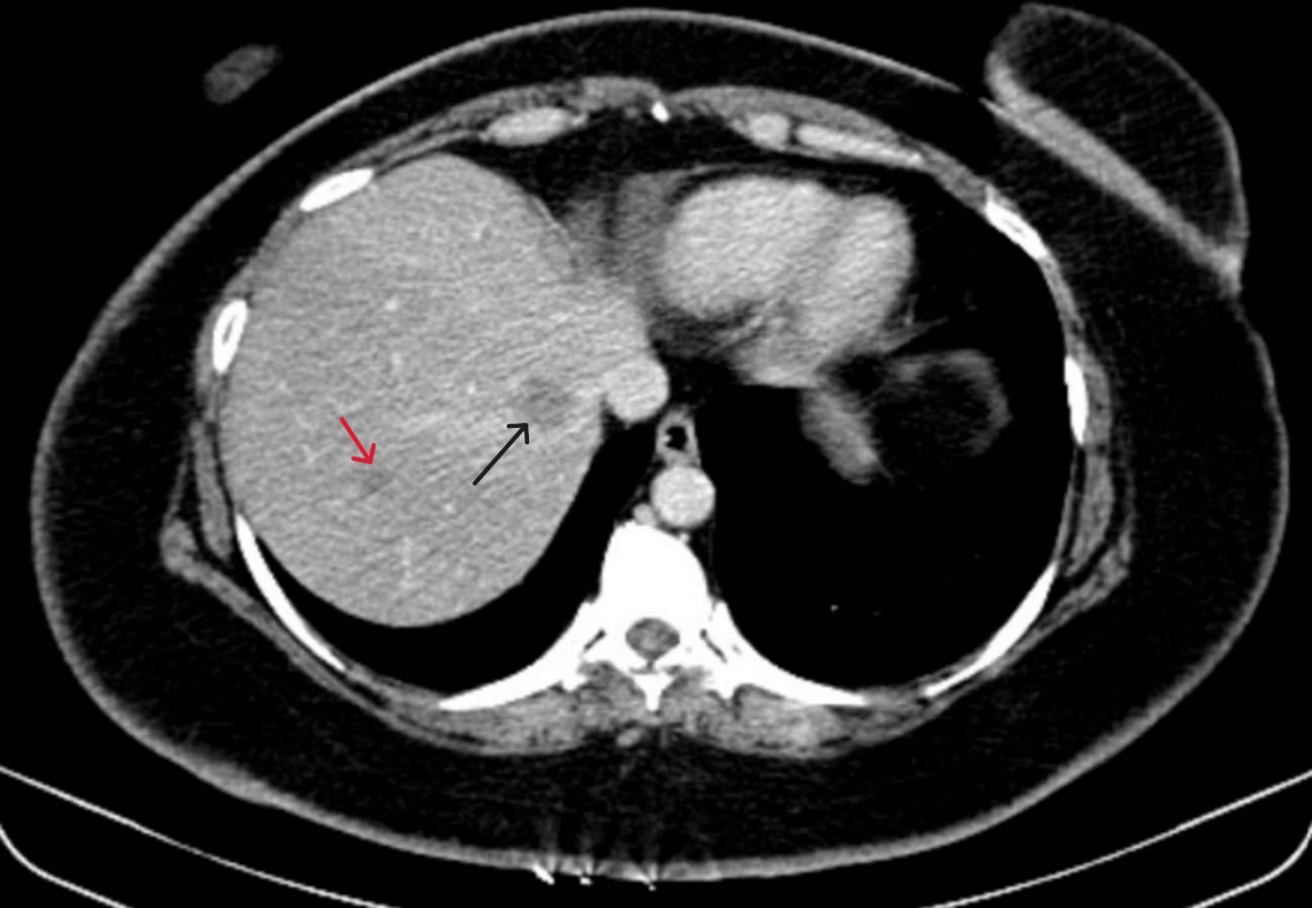
Contrast-enhanced computed tomography scan hepatic venous phase axial cut showing a lesion in segment 7 (red arrow) and segment 8 (black arrow) of the liver.

**Figure 5:**
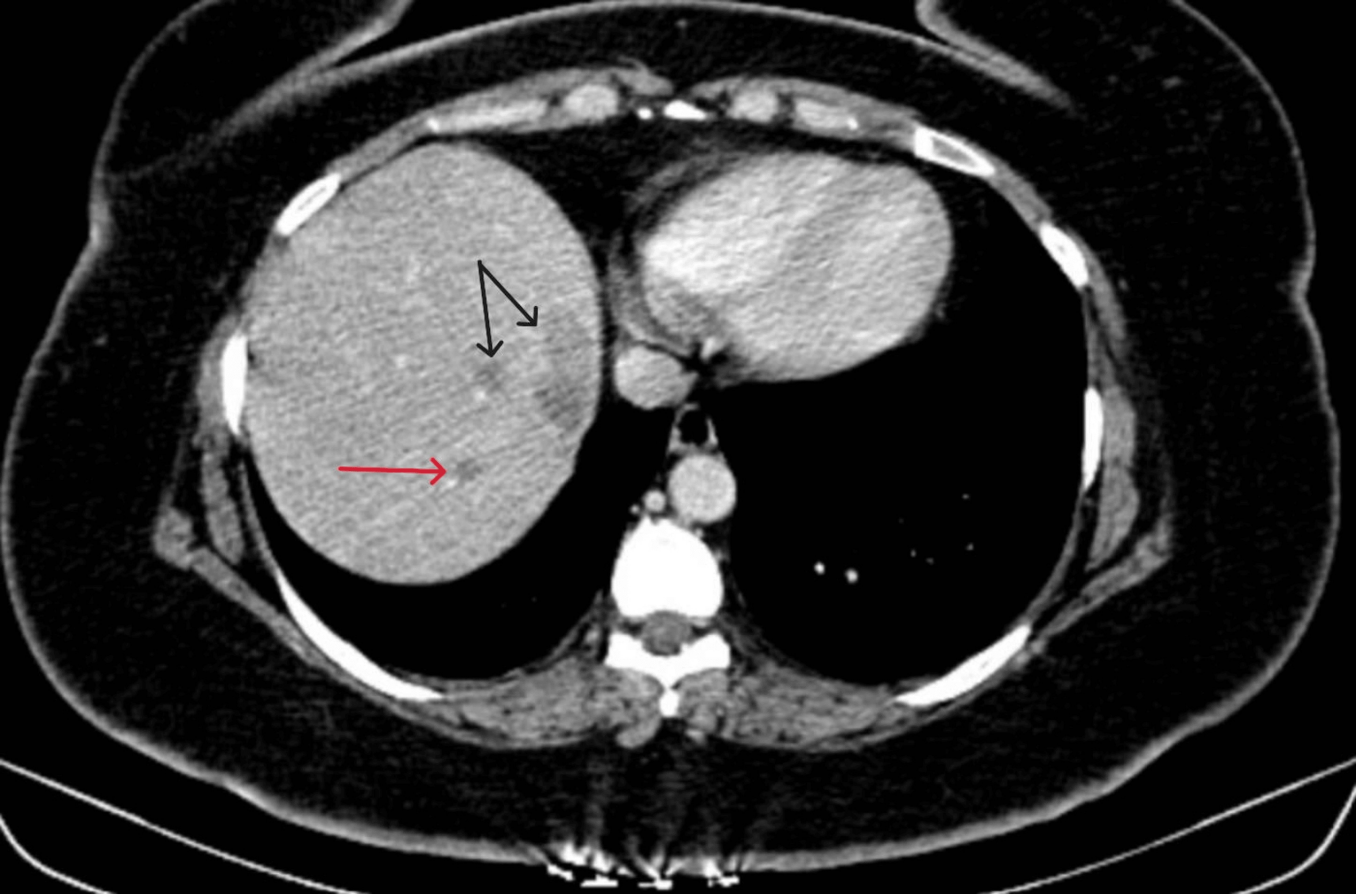
Contrast-enhanced computed tomography scan hepatic venous phase axial cut showing lesions in segments 7 (red arrow) and 8 (black arrows) of the liver.

Subsequently, in 2024, imaging revealed suspicious liver lesions on PET-CT, which were further confirmed by a Magnetic Resonance Imaging (MRI) liver protocol and biopsy to be metastases from ACC. She was asymptomatic for the same. She had multiple lesions in the right liver and a separate lesion in hepatic segment II, as shown in Figures 1-5.

**Figure 6:**
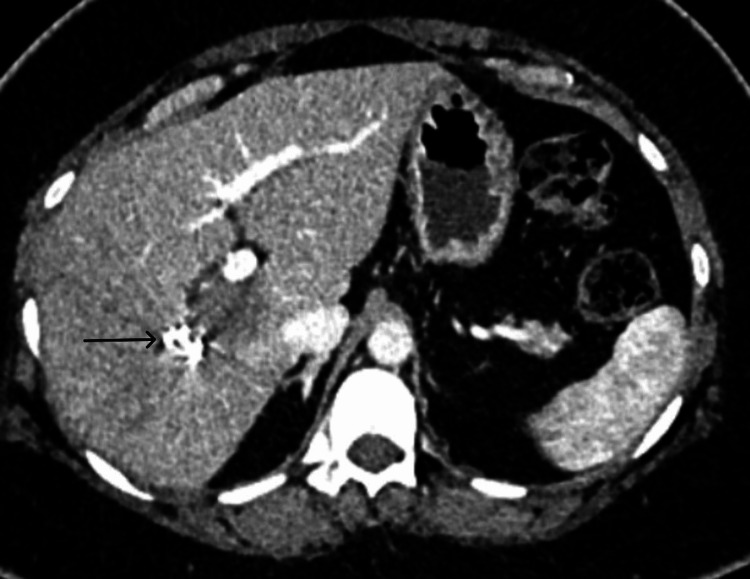
Contrast-enhanced computed tomography scan portal phase axial cut showing post-embolization hypertrophy of the left hemiliver. The embolization coils (black arrow) can be seen in the right portal vein, creating an artifact in the scan image.

Due to inadequate future liver remnant for a right hemi-hepatectomy, a right portal vein embolization was advocated to improve hepatic reserve. The left liver hypertrophy was significant at four weeks, and a kinetic growth rate of 3% suggested adequate functional reserve, as shown in Figure 6.

**Figure 7:**
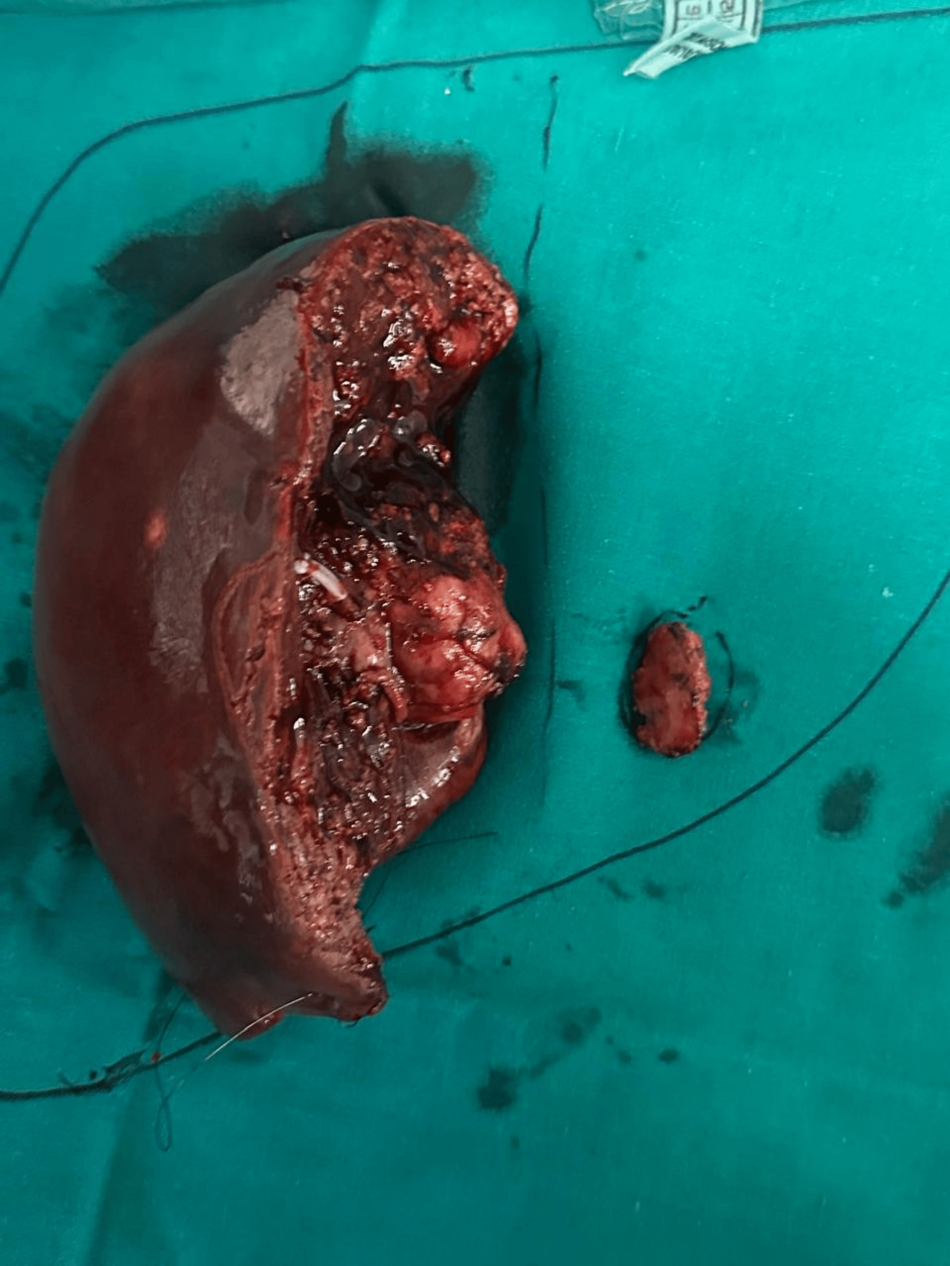
Postoperative specimen pic showing the right hemi-hepatectomy specimen with caudate excision and the segment 2 lesion.

She underwent a right hemi-hepatectomy with caudate and segment II metastatectomy, which resulted in an R0 resection, as shown in Figure 7.

Her postoperative recovery was uneventful, and she was discharged on postoperative day 7. She is disease-free at the six-month follow-up. The diagnosis of ACC was confirmed on histopathology showing cribriform and tubular architecture with a solid component of around 24% and perineural invasion, with immunohistochemistry positive for CD117, SMA, and S100.

## Discussion

ACC is an uncommon malignancy, accounting for approximately 1%-2% of all head and neck cancers and about 10% of all salivary gland neoplasms. Among the salivary glands, ACC can affect both major and minor glands, with a slight predilection for the latter. The parotid gland is involved in most cases among the major salivary glands. It typically affects adults in the age group of 40 and 60 years, with a slight female predominance [1,2].

Clinically, patients often present with a slow-growing, painless mass, though peri-neural invasion can lead to facial nerve dysfunction. Pathologically, ACC is classified into three histological subtypes: cribriform, tubular, and solid, with the solid variant being associated with the worst prognosis. Early diagnosis and multidisciplinary management are crucial to improve outcomes [1-3]. ACC typically shows a cribriform or mixed histology with perineural invasion. Although it predominantly metastasizes hematogenously, cervical lymph node involvement occurs in only 5%-15% of cases, making nodal spread relatively uncommon [1,2,3].

The primary treatment modality for ACC is surgical resection with clear margins. Adjuvant radiation therapy is commonly employed to enhance local control, particularly in cases with positive margins or perineural invasion. The role of chemotherapy in ACC is limited, as the tumor exhibits relative resistance to conventional chemotherapeutic agents. Chemotherapy may be considered in cases of advanced, unresectable, or metastatic disease, but its efficacy is generally modest. Long-term outcomes are characterized by high rates of local recurrence and distant metastasis even decades after curative treatment of the primary tumor in its early stages [1,4,5].

The management of neck node metastasis in ACC typically involves surgical intervention, such as neck dissection, especially when there is clinical or radiological evidence of nodal involvement. Adjuvant radiation therapy may be considered to improve local control. However, the role of elective neck dissection in clinically node-negative patients remains controversial due to the relatively low incidence of lymph node metastasis in ACC compared to other head and neck malignancies [4,5,6].

ACC is notorious for its propensity for hematogenous spread, leading to distant metastases. The lungs are the most common site of distant metastasis, followed by bone and, less frequently, the liver. Approximately 30%-40% of ACC patients develop distant metastasis within 10-15 years following curative-intent treatment, most commonly to the lungs. Liver metastasis from ACC is rare, with limited cases reported in the literature [5,6].

A comparative analysis of reported cases of ACC with liver metastasis is presented in Table 1 [3,5-8].

**Table 1:** Comparative analysis of published adenoid cystic carcinoma (ACC) cases with liver metastasis. RFA, radiofrequency ablation

Case	Age/Sex	Primary site	Time to liver metastasis	Treatment of liver metastasis	Outcome	Reference
1	32/F	Parotid gland	5 years	Right hemi-hepatectomy + metastatectomy	Alive on six-month follow-up	Present case
2	48/M	Parotid gland	5 years	Surgical resection	Died at 20 months	[3]
3	60/F	Parotid gland	3 years	No treatment - patient refused palliative chemotherapy	Died at 21 months	[5]
4	61/F	Minor salivary gland	4 years	Palliative chemotherapy	Died at 16 months	[6]
5	57/F	Palate	Not specified	Hepatic resection + RFA	Alive at 12 months	[7]
6	29/F	Parotid Gland	5 years	3 cycles of chemotherapy followed by liver resection	Alive at 12 months	[8]

Table 1 highlights the variability in patient demographics, primary tumor sites, time to liver metastasis, treatment approaches, and outcomes in ACC cases with liver metastasis. It underscores the importance of individualized treatment planning and the potential benefits of surgical intervention in selected patients with liver metastasis [6,7,8].

The median survival time of patients with ACC liver metastases is approximately 14 months, with one-, two-, and three-year survival rates of 55.8%, 28.5%, and 15.2%, respectively. Local treatments, such as surgical resection or radiofrequency ablation (RFA), may prolong survival in patients with isolated liver metastases [8,9]. 

Surveillance for ACC mandates a lifelong, multimodal approach due to its propensity for delayed distant metastases. Routine clinical follow-up with head and neck examination and cranial nerve assessment is recommended every three to six months for the first two years and annually thereafter. MRI of the head and neck is preferred for local and perineural recurrence, while annual chest CT is indicated for pulmonary metastasis. In patients with high-risk features, such as prior distant metastases, solid or high-grade histology, perineural invasion, close or positive margins, and recurrence-annual liver imaging with contrast-enhanced MRI or ultrasound is warranted. PET-CT may be used selectively based on clinical suspicion [7,8,9].

## Conclusions

ACC with liver metastasis is a rare and challenging clinical scenario, with limited reported cases in the literature. The need for long-term follow-up remains crucial due to the potential for delayed metastasis, highlighting the importance of early detection and tailored treatment strategies. This case emphasizes the importance of individualized care and close monitoring for patients with ACC, particularly those at risk of distant metastases. Metastatic ACC is challenging to treat due to its resistance to conventional therapies. Liver resection can be considered a feasible treatment option in select cases with isolated metastasis, potentially offering improved survival outcomes.

## References

[1] Kokemueller H, Eckardt A, Brachvogel P, Hausamen JE (2004). Adenoid cystic carcinoma of the head and neck--a 20 years experience. Int J Oral Maxillofac Surg.

[2] van der Wal JE, Becking AG, Snow GB, van der Waal I (2002). Distant metastases of adenoid cystic carcinoma of the salivary glands and the value of diagnostic examinations during follow-up. Head Neck.

[3] Bradley PJ (2004). Adenoid cystic carcinoma of the head and neck: a review. Curr Opin Otolaryngol Head Neck Surg.

[4] Mendenhall WM, Morris CG, Amdur RJ, Werning JW, Hinerman RW, Villaret DB (2004). Radiotherapy alone or combined with surgery for adenoid cystic carcinoma of the head and neck. Head Neck.

[5] Harish K, Mangala Gouri SR (2004). Adenoid cystic carcinoma of the parotid metastasizing to liver: case report. BMC Cancer.

[6] Chen AM, Bucci MK, Weinberg V (2006). Adenoid cystic carcinoma of the head and neck treated by surgery with or without postoperative radiation therapy: prognostic features of recurrence. Int J Radiat Oncol Biol Phys.

[7] Coca-Pelaz A, Rodrigo JP, Bradley PJ (2015). Adenoid cystic carcinoma of the head and neck--an update. Oral Oncol.

[8] Zemni I, Tounsi N, Bouraoui I (2019). A single liver metastasis from adenoid cystic carcinoma of the parotid gland: case report. J Investig Med High Impact Case Rep.

[9] Zheng Y, He Y, Wu F, Liu M, Wang L, Wu J (2020). Possible local treatment for liver metastases of adenoid cystic carcinoma (ACC): single-centre experience and literature review. Transl Cancer Res.

